# Questions and Answers

**Published:** 1887-03-26

**Authors:** 


### QUESTIONS AND ANSWERS.

All questions should be addressed to Mr. Howard J. Collins, Secretary, the Hospitals Association, Norfolk House, Norfolk Street, Strand, W.C.

### MATRONS AND NURSES FOR BRITISH HOSPITALS IN INDIA.

Miss K. Kearney, Matron of the Corporation Hospital, Bootle-cum-Linacre, wishes to know from what source the British Hospitals in India are supplied with nurses and matrons.

*** Army hospitals for British troops in India possess no staff of nurses at present. Matrons and nurses for other hospitals in India, when required, are not engaged through the India Office, nor is there any official information as to the source from which they are obtained. It is understood, however, that the Lady Superior and nurses employed in the Calcutta hospitals were obtained by the trustees of the Canning Home for Nurses from Clewer.

### BIPEDS *v.* QUADRUPEDS: STRETCHER-DRILL.

H. J. C. writes :—"In your article in The Hospital of the 5th inst., I observe you say, *re* carrying the patient, 'but the men must take care to keep in step,' whereas, in the stretcher-drill of the St. John's Ambulance Association (of which I have two certificates), we are taught that No. 1, at the head of the stretcher, should step off with the *left* foot, and No. 2, at the foot of the stretcher, having back to the patient, with the *right* foot, both taking short steps of about 20 inches, and with the knees bent. Many, probably, as well as myself would like to know the reason of this different advice, or a line that the meaning was 'in step,' *i.e.*, regular."

*** There is no "different advice" intended. In carrying an injured man on a stretcher it is imperative that there should be as little movement of the stretcher as possible. For the attainment of this object it is absolutely necessary to keep in step. It is also desirable that No. 1 should step off with the *left* foot and No. 2. with the *right*. By this means a still greater degree of steadiness is gained by the stretcher. A reference to the elementary principles of mechanics will show at once why this must be so; but an easy example may be observed any day in the streets. If H. J. C., or others in a similar difficulty, will watch the movements of a horse in walking or trotting (slowly walking, if possible), he will see that the animal walks *cross-footed*, so to speak: that is, the left hind-leg follows the right forefoot, and the right hind-leg the left forefoot. The horse's back is thus almost perfectly steady; whereas, if the animal were to walk with the legs of the *same side following each other*, a slightly *rolling* movement would be observable in the body, which, if he were trotting, would make the pace very uncomfortable for the rider.

### THE COST OF ENDOWING A COTTAGE HOSPITAL BED.

Miss E. P. Smith writes from Mansfield and Mansfield Woodhouse District Hospital:—"I trust you will not consider me a too frequent correspondent, but a gentleman has asked me to ascertain for him what the cost of endowing a bed in a Cottage Hospital would be? So I shall be glad if you will kindly tell me through The Hospital. Our reports are not printed yet; as soon as they are, I will send you one."

*** £1,250, *i.e.*, £50 per bed per annum on a 4 per cent. investment.

